# FACTORS ASSOCIATED WITH HOME HEALTH CARE VERSUS SKILLED NURSING FACILITY REFERRAL FOLLOWING HOSPITAL DISCHARGE

**DOI:** 10.1093/geroni/igae098.3843

**Published:** 2024-12-31

**Authors:** Natalie Turner, Amber Sabbatini

**Affiliations:** University of Washington, Seattle, Washington, United States; University of Washington, Seattle, Washington, United States

## Abstract

Among post-acute services, home health care has markedly increased following hospitalization. Home health care is less expensive compared to other post-acute services, namely skilled nursing facilities, and is more aligned with older adults’ desire to remain at home. The objective of this study was to identify individual, hospital, and community factors associated with home health care referral among Medicare beneficiaries. Multivariate logistic regressions with hospital-level fixed effects were used to estimate the patient, hospital and community-level factors associated with home health care referral among Medicare beneficiaries at hospital discharge. Older age, being female, and dual enrolled beneficiary status were associated with decreased odds of home health care referral. Beneficiaries living in rural compared to urban settings had higher odds of home health care referral. Communities with a higher proportion of people of color had a lower odds of home health referral, while having a greater percentage of residents living in poverty was associated with higher odds. The decision to refer to home health care was largely driven by patient-level factors. However, the racial and ethnic composition of a community as well its economic status and rurality may affect the odds of a home health care referral beyond both the patient characteristics and the specific hospital in which they were treated. Ensuring equitable access to home health care necessitates increased investment in care infrastructure and policies that dismantle structural barriers to preferred post-acute care settings.

